# Deep Learning Approaches to Forecast Physical and Mental Deterioration During Chemotherapy in Patients with Cancer

**DOI:** 10.3390/diagnostics15080956

**Published:** 2025-04-09

**Authors:** Joseph Finkelstein, Aref Smiley, Christina Echeverria, Kathi Mooney

**Affiliations:** 1 Department of Biomedical Informatics, The University of Utah, Salt Lake City, UT 84108, USA; joseph.finkelstein@utah.edu; 2 College of Nursing, The University of Utah, Salt Lake City, UT 84108, USA; christina.echeverria@nurs.utah.edu (C.E.); kathi.mooney@nurs.utah.edu (K.M.)

**Keywords:** chemotherapy symptom prediction, LSTM, CNN, GRU models, AI-driven symptom monitoring, oncology symptom management, digital health, AI

## Abstract

**Background/Objectives**: Predicting symptom escalation during chemotherapy is crucial for timely interventions and improved patient outcomes. This study employs deep learning models to predict the deterioration of 12 self-reported symptoms, categorized into physical (e.g., nausea, fatigue, pain) and mental (e.g., feeling blue, trouble thinking) groups. **Methods**: The analytical dataset comprises daily self-reported symptom logs from individuals undergoing chemotherapy. To address class imbalance—where 84% of cases showed no escalation—symptoms were grouped into intervals of 3 to 7 days. Convolutional Neural Networks (CNNs), Long Short-Term Memory (LSTM), and Gated Recurrent Unit (GRU) models were trained on 80% of the data and evaluated on the remaining 20%. **Results**: Results showed that 3-day intervals yielded the best predictive performance. CNNs excelled in predicting physical symptoms, achieving 79.2% accuracy, 84.1% precision, 78.8% recall, and an F1 score of 81.4%. For mental symptoms, GRU outperformed other models, with an accuracy of 77.2%, precision of 71.6%, recall of 62.2%, and an F1 score of 66.6%. Performance declined for longer intervals due to reduced temporal resolution and fewer training samples, though CNNs and GRU remained relatively stable. **Conclusions**: The findings emphasize the advantage of categorizing symptoms for more tailored predictions and demonstrate the potential of deep learning in forecasting symptom escalation. Integrating these predictive models into clinical workflows could facilitate proactive symptom management, allowing timely interventions and enhanced patient care during chemotherapy.

## 1. Introduction

Effective symptom management is a critical component of oncology care, directly influencing quality of life and treatment adherence. Individuals undergoing chemotherapy often experience a range of symptoms, including pain, fatigue, nausea, vomiting, and cognitive disturbances, which vary based on cancer type, disease stage, and treatment regimen [1]. According to the National Cancer Institute, between 20% and 50% of those receiving treatment experience pain, while fatigue is reported in 14% to 96% of cases [2]. Addressing these symptoms requires a multidisciplinary approach integrating evidence-based strategies, digital health technologies, and machine learning techniques [3].

Studies have demonstrated that proactive symptom monitoring improves outcomes by enabling early interventions and reducing the severity of treatment-related side effects [4,5,6]. Chemotherapy, while a cornerstone of cancer treatment, often leads to severe adverse effects that compromise well-being, including extreme fatigue, gastrointestinal issues, immune suppression, and neurotoxicity [7]. These side effects frequently contribute to treatment nonadherence, which impacts overall survival and disease progression [1]. Recent studies indicate that integrating self-reported symptoms into clinical workflows enhances symptom detection, extends survival, and improves overall care [8].

The rapid advancement of digital health technologies has facilitated real-time symptom monitoring, allowing automated symptom tracking and timely clinical interventions [9]. Telemonitoring systems have gained widespread acceptance among individuals with chronic conditions and seniors [10,11,12], providing a structured means for tracking symptom deterioration and alerting healthcare providers to potential complications [13,14]. The implementation of these digital systems has demonstrated significant potential in addressing the evolving and complex needs of individuals undergoing treatment, their caregivers, and healthcare providers [15]. However, a major challenge remains in utilizing self-reported outcomes to predict symptom escalation and determine the optimal timing for clinical intervention [16]. Traditional symptom management protocols often lack specificity, making it difficult for healthcare providers to decide when interventions are necessary [17,18].

### Literature Review

There is a crucial demand for novel approaches that provide seamless and ongoing support to improve symptom oversight in patients with cancer. To address these limitations, machine learning (ML) and deep learning (DL) models have emerged as powerful tools for predicting symptom escalation [19,20]. Among these, Long Short-Term Memory (LSTM) networks and Convolutional Neural Networks (CNNs) have demonstrated substantial promise in analyzing longitudinal self-reported data [21,22,23,24]. LSTM models effectively capture temporal dependencies in sequential symptom data, identifying early patterns indicative of impending deterioration [25,26,27,28]. In contrast, CNNs leverage spatial feature extraction to identify patterns within structured symptom datasets, offering an alternative approach to symptom trajectory modeling [29,30,31].

Several studies have explored the potential of deep learning to predict symptom progression based on self-reported outcomes [32,33,34]. This study builds on prior research by investigating the predictive capabilities of CNN, LSTM, and Gated Recurrent Unit (GRU) models in forecasting chemotherapy-related symptom escalation. By analyzing self-reported symptom data across varying time intervals (3 to 7 days), this study evaluates the effectiveness of each model in detecting significant symptom escalation. The findings aim to enhance symptom prediction accuracy, allowing healthcare providers to intervene earlier and personalize treatment strategies.

By integrating deep learning models into digital health systems, clinicians can receive real-time alerts on symptom escalation, enabling proactive management and potentially improving overall treatment outcomes. This study makes several key contributions to the growing field of AI-driven healthcare: (1) it evaluates and compares the performance of three deep learning models—CNN, LSTM, and GRU—using high-frequency, real-world symptom data from chemotherapy patients; (2) it explores varying time intervals and a sliding-window approach to optimize temporal prediction; and (3) it distinguishes between physical and mental symptoms to support more targeted interventions. This research contributes to the growing field of AI-driven healthcare by demonstrating how predictive models can be leveraged to optimize symptom monitoring, reduce distress, and improve adherence to therapy.

## 2. Methods

### Data Collection and Preprocessing

This study utilized data from the Symptom Care at Home Study [35], comprising a total of 26,599 symptom records collected from individuals undergoing chemotherapy. The dataset included a diverse range of participants with variations in age, gender, ethnicity, and socioeconomic status. Table 1 presents a comprehensive demographic summary of the study population. The most common diagnoses are breast cancer (156 participants), lung cancer (61 participants), and ovarian cancer (36 participants), followed by other conditions such as colorectal (26), pancreatic (21), and head and neck cancers (13).

To ensure consistent and reliable data collection, participants were instructed to record their symptoms daily using a technology-based system. This allows continuous tracking of symptom progression. The dataset included 12 key symptoms, categorized into two primary groups:

1. Physical Symptoms: Nausea/vomiting, sore mouth, diarrhea, fatigue, hair loss/weight change, pain/discomfort, numbness/tingling, and fever-related distress. These symptoms reflect physiological responses to chemotherapy and cancer-related effects.
2. Mental Symptoms: Trouble thinking, feeling blue or nervous/anxious, and trouble sleeping. These symptoms represent cognitive and emotional challenges associated with treatment.

For each symptom, two quantitative metrics were recorded:

1. Severity (rated from 1 to 10, where 10 represents the most intense experience).
2. Distress (rated from 1 to 10, indicating the level of emotional burden associated with the symptom).

If a symptom was not reported on a given day, both metrics were recorded as zero. Fever was an exception, where only distress values were recorded. A daily symptom burden score was computed by summing all severity and distress values, resulting in a total possible score range of 0 to 230 per day.

The dataset included 349 participants, each undergoing an average of three chemotherapy cycles, with a median participation period of 81 days. Missing values in the dataset, particularly for symptom severity and distress, were addressed using linear interpolation, which estimated values based on adjacent days’ records. This approach ensured the continuity and reliability of data without introducing artificial fluctuations.

Due to the highly imbalanced nature of the dataset, where 84% of recorded instances indicated no symptom escalation, we implemented a resampling strategy to enhance the model’s ability to detect meaningful symptom variations. Symptom data were aggregated into multi-day intervals (3 to 7 days), thereby reducing the number of zero scores while preserving temporal progression. This interval-based approach enabled a more balanced distribution of data, allowing for improved model training and detection of symptom escalation trends.

## 3. Resampling and Interval-Based Data Structuring

To optimize model performance, symptom data were grouped into intervals of 3, 4, 5, 6, or 7 days. Within each interval, symptom scores were averaged to capture overall symptom trends, rather than day-to-day fluctuations, while maintaining temporal relationships.

To ensure statistical robustness, only individuals with at least (*n* × 7) days of continuous data were included in the analysis, where *n* represents the interval length. For example, for a 3-day interval, participants needed at least 21 days of data to be included in the model. Individuals with insufficient records were excluded to ensure uniformity in training and testing datasets. Shorter intervals (e.g., 3 days) retained high temporal resolution, but increased data variability and longer intervals (e.g., 7 days) provided smoother trends but potentially masked short-term symptom escalations.

A binary classification variable was assigned for each interval: 1 (escalation detected) if the total symptom burden score increased beyond a predefined threshold and 0 (no escalation) if the symptom burden remained stable or decreased.

The predictive framework used data from past intervals (e.g., intervals 1–3) to forecast symptom escalation in the next interval (e.g., interval 4). This sliding-window approach ensured that the model learned from sequential patterns in symptom progression.

## 4. Deep Learning Model Architecture

Deep learning models were selected for their strong performance in modeling complex and temporal relationships in patient-reported outcome data. Long Short-Term Memory (LSTM) and Gated Recurrent Unit (GRU) are types of recurrent neural networks (RNNs) designed to capture sequential dependencies and long-term patterns across time-series data, making them well-suited for predicting symptom progression. Convolutional Neural Networks (CNNs), although originally developed for image analysis, have shown strong performance in extracting features from structured time-series data due to their ability to recognize localized patterns. These models were chosen to evaluate different learning mechanisms and identify the most effective approach for forecasting physical and mental symptom escalation during chemotherapy. The architectural details of each deep learning model are described in the following subsections.

### 4.1. Long Short-Term Memory (LSTM)

LSTM networks are designed to capture longitudinal dependencies within sequential data, making them well-suited for symptom prediction. The architecture included the following:

- A 50-unit LSTM layer to process temporal trends.
- A dropout layer (0.2 probability) to mitigate overfitting.
- A fully connected (dense) layer with sigmoid activation for binary classification.
- Binary cross-entropy loss function with the Adam optimizer for efficient learning.

### 4.2. Convolutional Neural Network (CNN)

CNNs were used to detect localized patterns in symptom data, recognizing critical escalation features across intervals. The model structure comprised the following:

- A 1D convolutional layer (64 filters, kernel size = 3) for feature extraction.
- Max-pooling and flattening layers to reduce data dimensionality.
- Dense layers with ReLU activation for feature refinement.
- A dropout layer for regularization.
- A sigmoid-activated output layer for binary classification.

### 4.3. Gated Recurrent Unit (GRU)

GRUs are an efficient alternative to LSTMs, designed to process sequential patterns with fewer computational requirements. The model included the following:

- A 50-unit GRU layer for learning temporal dependencies.
- Dropout regularization to prevent overfitting.
- A final sigmoid-activated layer for binary classification.

Each model was trained using 80% of the dataset, while 20% was reserved for evaluation. Performance was assessed using key classification metrics, including accuracy, precision, recall, F1 score, and area under the receiver operating characteristic curve (AUC).

**Figure 1:**
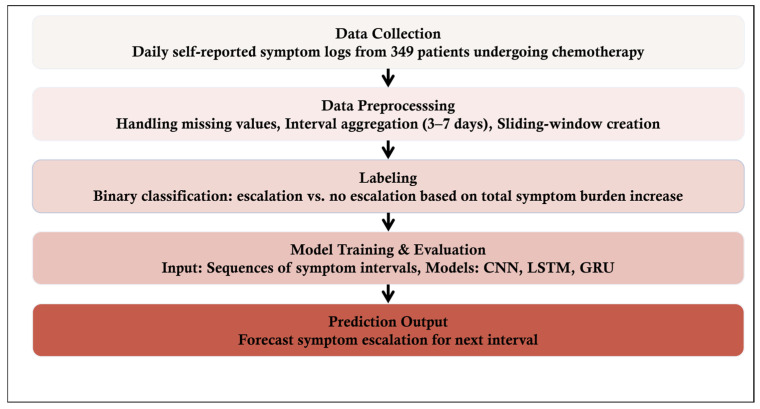
Workflow of the proposed deep learning-based framework for forecasting symptom escalation in chemotherapy patients.

An overview of the proposed symptom prediction framework is illustrated in Figure 1.

## 5. Ethical Considerations

This study was conducted in compliance with ethical research standards and received Institutional Review Board (IRB) approval from the University of Utah (IRB protocol: IRB_00017472).

## 6. Results

The performance of deep learning models (CNN, LSTM, and GRU) was assessed for predicting physical and mental symptom escalation in chemotherapy patients. The dataset was analyzed using different interval lengths (3, 4, 5, 6, and 7 days), with model evaluation metrics including accuracy, precision, recall, F1 score, and AUC (area under the curve). A summary of the results across models and time intervals is provided in Table 2, Table 3, Table 4, Table 5, Table 6 and Table 7.

For predicting physical symptom escalation, the 3-day interval consistently demonstrated the highest predictive accuracy across all models. The CNN, LSTM, and GRU models exhibited distinct strengths across the assessed metrics (Table 2, Table 3 and Table 4). Among them, GRU demonstrated the highest overall performance, achieving an accuracy of 80.05% and an F1 score of 82.50% at the 3-day interval, making it the most effective model for short-term symptom prediction.

For mental symptoms, Table 5, Table 6 and Table 7 present the performance metrics of each model across different interval lengths. Among the models, CNN exhibited a slight advantage in AUC, achieving 77.26%, whereas LSTM outperformed the others in recall (63.45%) and F1 score (67.23%) at the 3-day interval, indicating its effectiveness in capturing symptom escalation patterns over short-term periods.

All models demonstrated comparable effectiveness in predicting both physical and mental symptom escalation, with their highest performance metrics observed at the 3-day interval. As the interval length increased, a decline in predictive performance was evident across all models. While CNN, LSTM, and GRU exhibited slight variations in specific metrics, their overall performance remained relatively similar, suggesting that no single model consistently outperformed the others across all evaluation criteria.

## 7. Discussion

The findings of this study highlight the potential of deep learning models—particularly LSTM, CNN, and GRU—in predicting symptom escalation among chemotherapy patients. Model performance was strongest when using shorter intervals (*n* = 3 days). However, as the interval length increased, accuracy and recall declined across all models, though precision remained relatively stable. These results suggest that shorter intervals are more effective for tracking rapid symptom fluctuations, a common challenge in chemotherapy symptom management.

The comparative analysis of these models underscores their complementary strengths. While LSTMs and GRUs excel at learning sequential dependencies in symptom progression, CNNs enhance feature extraction, potentially improving prediction accuracy in structured patient-generated data. This multi-model approach can be adapted for various clinical applications, particularly in continuous symptom monitoring scenarios where early intervention is critical. Implementing these models in digital health systems could enable real-time symptom tracking and automated alerts for healthcare providers, facilitating proactive interventions for patients at high risk of symptom escalation. This aligns with the growing trend of integrating AI into oncological care, where early symptom management has been shown to improve clinical outcomes and patient satisfaction.

A key challenge in this study was the highly imbalanced dataset, with 84% of recorded symptom instances showing no escalation. To address this, symptom data were aggregated into multi-day intervals, reducing the dominance of zero scores and improving model training. However, the observed decline in predictive performance for longer intervals suggests that additional strategies are needed to manage class imbalance effectively. Future research should explore techniques such as oversampling, synthetic data generation, and advanced resampling strategies to further enhance the models’ ability to detect symptom escalation across varying time frames.

Although our study included 349 participants, the dataset encompassed over 26,000 daily symptom entries, offering substantial longitudinal data for model training and evaluation. The application of temporal aggregation and sliding-window techniques enabled efficient use of this data by capturing dynamic symptom trends over time. However, we acknowledge that a larger and more demographically diverse participant pool could enhance the generalizability and robustness of the models.

This study focused on deep learning models due to their proven effectiveness in capturing temporal dependencies and complex nonlinear patterns in sequential health data. These architectures are particularly well-suited for modeling symptom progression over time, which is critical in the context of chemotherapy monitoring. While traditional machine learning models such as Random Forest and Support Vector Machines have shown utility in various clinical prediction tasks, they are often limited in their ability to handle time-series data without extensive feature engineering. Future work will include benchmarking against these classical models to provide a more comprehensive comparison and assess the added value of deep learning approaches in symptom prediction.

Our findings align closely with previous studies highlighting the potential of deep learning in predictive modeling of sequential patient-generated data [31,32,33,34,36]. Despite the promising results, several limitations should be considered. This study relied on self-reported symptom data, which may be subject to biases such as underreporting or inconsistencies in daily logging. Future research could integrate objective physiological markers (e.g., heart rate, activity levels) [37,38] and data from electronic health records (EHRs) [24,39] to supplement self-reported symptoms, enhancing prediction accuracy and robustness. Enrichment of self-reported symptoms by real-world data extracted from electronic health records (EHRs) can help improve the predictive model performance [40,41]. Clinical decision support for early identification of cancer symptom deterioration embedded into EHRs can potentially improve the quality of life of cancer patients undergoing chemotherapy [42,43]. Additionally, this study focused on chemotherapy patients, and further investigation is needed to assess the generalizability of these models across different cancer types and treatment regimens and broader patient populations [44].

## 8. Conclusions

This study demonstrates the effectiveness of deep learning models—CNN, LSTM, and GRU—in predicting symptom escalation in chemotherapy patients. By leveraging AI-driven symptom monitoring, healthcare providers can deliver more personalized, proactive care, potentially improving patient outcomes and quality of life. By integrating these models into digital health platforms or electronic health records, clinicians could receive real-time alerts, enabling timely interventions that may prevent complications, improve patient comfort, and support treatment adherence. Future work should focus on refining these models for extended time intervals, addressing data imbalance more effectively, and exploring their applicability across diverse clinical settings to maximize their impact on patient care.

## Figures and Tables

**Table 1:** This table provides an overview of the demographic characteristics of the 349 participants included in the study.

Category	Count	Percentage
Total Participants	349.0	100%
Gender—Female	263.0	75.4%
Gender—Male	86.0	24.6%
Age—Mean	55.8	-
Age—Median	56.6	-
Age—Min	24.0	-
Age—Max	85.0	-
Race—White	290.0	82.96%
Race—Black	40.0	11.45%
Race—Asian	5.0	1.4%
Race—Unknown/Other	5.0	1.4%
Marital Status—Married	184.0	52.7%
Marital Status—Single	112.0	32.1%
Marital Status—Other	53.0	15.2%
Education—Some College/Technical	125.0	35.8%
Education—High School Graduate	87.0	24.9%
Education—Bachelor’s Degree	66.0	18.9%
Education—Graduate/Professional Degree	22.0	6.3%
Education—Less than High School	49.0	14.1%
Employment—Full-time (31+ h)	73.0	20.9%
Employment—Part-time (11–30 h)	55.0	15.8%
Employment—Minimal (1–10 h)	18.0	5.2%
Employment—Unemployed/Retired	203.0	58.1%
Income—USD 10,000-USD 19,999	82.0	23.5%
Income—USD 30,000-USD 39,999	64.0	18.2%
Income—USD 40,000-USD 49,999	54.0	15.4%
Income—USD 50,000-USD 69,999	47.0	13.5%
Income—Below USD 10,000 or Above USD 70,000	102.0	29.4%
Insurance—Medicaid/Medicare	165.0	47.3%
Insurance—Private	133.0	38.2%
Insurance—Uninsured	51.0	14.5%
Diagnosis—Breast Cancer	109.0	31.2%
Diagnosis—Lung Cancer	70.0	20.1%
Diagnosis—Ovarian Cancer	54.0	15.5%
Diagnosis—Other Malignancies	116.0	33.2%
Stage at Diagnosis—Stage IV	147.0	42.1%
Stage at Diagnosis—Stage III	103.0	29.5%
Stage at Diagnosis—Stage II	66.0	18.9%
Stage at Diagnosis—Stage I	33.0	9.5%

**Table 2:** CNN performance for physical symptoms.

Interval	Accuracy	Precision	Recall	F1 Score	AUC
3	79.22%	83.74%	79.41%	81.51%	83.08%
4	77.02%	82.76%	79.18%	80.93%	81.89%
5	73.98%	81.47%	77.60%	79.49%	77.29%
6	76.59%	82.84%	82.35%	82.60%	80.20%
7	73.17%	83.50%	75.17%	79.12%	78.00%

**Table 3:** LSTM performance for physical symptoms.

Interval	Accuracy	Precision	Recall	F1 Score	AUC
3	79.22%	84.16%	78.81%	81.40%	83.76%
4	77.21%	83.04%	79.18%	81.06%	82.74%
5	74.55%	82.57%	77.10%	79.74%	78.45%
6	76.69%	82.58%	82.94%	82.76%	81.07%
7	74.59%	82.99%	78.50%	80.68%	78.70%

**Table 4:** GRU performance for physical symptoms.

Interval	Accuracy	Precision	Recall	F1 Score	AUC
3	80.05%	83.51%	81.53%	82.50%	83.77%
4	76.43%	83.26%	77.27%	80.15%	82.22%
5	73.33%	81.53%	76.22%	78.78%	77.45%
6	77.18%	83.68%	82.21%	82.94%	80.71%
7	74.35%	84.20%	76.40%	80.11%	78.48%

**Table 5:** CNN performance for mental symptoms.

Interval	Accuracy	Precision	Recall	F1 Score	AUC
3	77.36%	72.83%	60.64%	66.18%	77.26%
4	74.35%	71.68%	60.10%	65.38%	75.72%
5	70.65%	69.40%	61.45%	65.19%	72.98%
6	71.73%	69.52%	66.30%	67.87%	75.21%
7	70.33%	70.99%	63.00%	66.75%	72.96%

**Table 6:** LSTM performance for mental symptoms.

Interval	Accuracy	Precision	Recall	F1 Score	AUC
3	77.41%	71.49%	63.45%	67.23%	77.11%
4	74.28%	71.05%	61.07%	65.68%	75.76%
5	70.49%	69.68%	60.18%	64.59%	72.88%
6	71.73%	69.52%	66.30%	67.87%	75.03%
7	69.98%	69.84%	64.25%	66.93%	73.08%

**Table 7:** GRU performance for mental symptoms.

Interval	Accuracy	Precision	Recall	F1 Score	AUC
3	77.21%	71.65%	62.25%	66.62%	77.09%
4	74.28%	72.13%	58.97%	64.89%	75.62%
5	70.57%	69.11%	61.82%	65.26%	72.86%
6	71.53%	69.65%	65.20%	67.35%	75.01%
7	69.74%	69.46%	64.25%	66.75%	72.85%

## Data Availability

The data presented in this study are available on request from the corresponding author.
